# (3D) Bioprinting—Next Dimension of the Pharmaceutical Sector

**DOI:** 10.3390/ph17060797

**Published:** 2024-06-17

**Authors:** Anna Mihaylova, Dobromira Shopova, Nikoleta Parahuleva, Antoniya Yaneva, Desislava Bakova

**Affiliations:** 1 Department of Healthcare Management, Faculty of Public Health, Medical University of Plovdiv, 4000 Plovdiv, Bulgaria; desislavabakova@gmail.com; 2 Department of Prosthetic Dentistry, Faculty of Dental Medicine, Medical University of Plovdiv, 4000 Plovdiv, Bulgaria; dent.shopova@gmail.com; 3 Department of Obstetrics and Gynecology, Faculty of Medicine, Medical University of Plovdiv, 4000 Plovdiv, Bulgaria; n_nikoleta1986@abv.bg; 4 Department of Medical Informatics, Biostatistics and eLearning, Faculty of Public Health, Medical University of Plovdiv, 4000 Plovdiv, Bulgaria; yaneva.antonya@gmail.com

**Keywords:** 3D bioprinting, drug research and development, personalized medicine, pharmaceutical company, clinical trials, drug testing

## Abstract

To create a review of the published scientific literature on the benefits and potential perspectives of the use of 3D bio-nitrification in the field of pharmaceutics. This work was performed in accordance with the Preferred Reporting Items for Systematic Reviews and Meta-Analyses (PRISMA) guidelines for reporting meta-analyses and systematic reviews. The scientific databases PubMed, Scopus, Google Scholar, and ScienceDirect were used to search and extract data using the following keywords: 3D bioprinting, drug research and development, personalized medicine, pharmaceutical companies, clinical trials, drug testing. The data points to several aspects of the application of bioprinting in pharmaceutics were reviewed. The main applications of bioprinting are in the development of new drug molecules as well as in the preparation of personalized drugs, but the greatest benefits are in terms of drug screening and testing. Growth in the field of 3D printing has facilitated pharmaceutical applications, enabling the development of personalized drug screening and drug delivery systems for individual patients. Bioprinting presents the opportunity to print drugs on demand according to the individual needs of the patient, making the shape, structure, and dosage suitable for each of the patient’s physical conditions, i.e., print specific drugs for controlled release rates; print porous tablets to reduce swallowing difficulties; make transdermal microneedle patches to reduce patient pain; and so on. On the other hand, bioprinting can precisely control the distribution of cells and biomaterials to build organoids, or an Organ-on-a-Chip, for the testing of drugs on printed organs mimicking specified disease characteristics instead of animal testing and clinical trials. The development of bioprinting has the potential to offer customized drug screening platforms and drug delivery systems meeting a range of individualized needs, as well as prospects at different stages of drug development and patient therapy. The role of bioprinting in preclinical and clinical testing of drugs is also of significant importance in terms of shortening the time to launch a medicinal product on the market.

## 1. Introduction

With the continual advancement in scientific and technological progress, the realm of 3D printing is undergoing constant expansion, penetrating an array of industrial sectors. The pharmaceutical industry has recognized the manifold advantages and merits associated with the adoption of this technology [1]. The incorporation of 3D printing methodologies within the pharmaceutical domain broadens the horizons for the creation of innovative, economically viable, environmentally sustainable, customized, individualized, and multifaceted drug delivery platforms [2,3,4,5].

Researchers in this field are directing their efforts towards the exploration of the benefits offered by 3D printing technology in the realm of drug delivery systems. Concurrently, this technology facilitates the exploration of diverse avenues for personalized medicine, encompassing aspects such as dosage, composition, geometry, dimensions, porosity, release kinetics, and other pertinent parameters.

The production of personalized pharmaceuticals is poised to revolutionize the field of healthcare by tailoring therapeutic formulations to an individual’s genetic profile, medical history, and physiological characteristics. The realization of precision medicines owes its existence to the integration of groundbreaking 3D printing technology. In comparison to traditional manufacturing methods, 3D printing offers advantages such as rapid production, intricate design capabilities, and bioprinting capabilities. Within the pharmaceutical sector, manufacturers are crafting compositions through the layer-by-layer deposition of adaptable polymers, allowing for the creation of diverse geometries, thicknesses, release profiles, and conformable designs. As this innovative technology progresses, the development of patient-centric medical devices and pharmaceutical products must comply with stringent regulatory standards to enter the market. The focal point lies in elucidating the rationale behind personalized medicine. The integrated applications of personalized drug delivery and 3D printing devices encompass dose customization, formulation of multiple drugs, modification of release systems, and demographic-specific customization [6].

Advancements in three-dimensional (3D) printing techniques, coupled with the emergence of customized biomaterials, have facilitated the precise fabrication of biological components and intricate 3D structures in recent decades. Additionally, the remarkable expansion of 3D printing has unlocked new possibilities within the realm of pharmaceuticals, allowing for the creation of personalized drug screening and drug delivery systems tailored to individual patients. This marks a departure from traditional approaches that heavily rely on transgenic animal experiments and mass production. The synergy of 3D printing and the availability of printable and biocompatible materials has undeniably catalyzed the production of personalized products in recent years [7].

**Figure 1:**
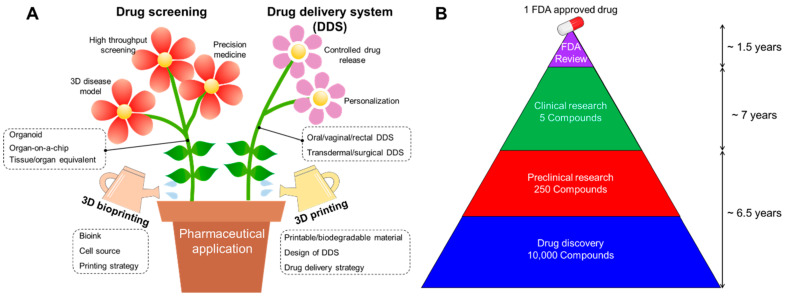
(**A**) Schematic illustration showing advances in pharmaceutical applications including drug screening and drug delivery system (DDS) using 3D printing and bioprinting technology. (**B**) Average drug administration schedule for one FDA-approved drug [9].

The ongoing advancements in tissue bioprinting are of paramount significance in advancing research objectives and supporting the pharmaceutical development process. The emergence of three-dimensional (3D) models has garnered substantial attention within the scientific community, owing to their capacity to faithfully replicate the intricate hierarchical tissue architecture and diverse composition observed in vivo. The successful integration of 3D biomodels holds the potential not only to enhance in vitro–in vivo correlations when compared to their 2D counterparts but also to supplant preclinical animal testing, which is accompanied by inherent limitations. Among the array of fabrication techniques, bioprinting, which encompasses various modalities such as extrusion, droplet, and laser bioprinting, has emerged as a viable, scalable, and dynamic approach for the creation of biomimetic tissue constructs. Although the ongoing technological progress necessitates more rigorous in silico design justifications, experimental in vitro studies demand increased confidence from the industry and a refinement of in vitro–in vivo correlations. The utilization of three-dimensional (3D) tissue models capable of mimicking both the spatial and chemical characteristics of natural tissues has demonstrated superior efficacy in drug screening compared to the conventional two-dimensional (2D) models. Nevertheless, the challenge of in vitro production of living tissues remains a hurdle in realizing the full potential of 3D models [8]. Recent advancements in bioprinting offer a valuable tool for the creation of biomimetic constructs, which can find applications across various stages of research aimed at the development of novel pharmaceuticals, as shown in Figure 1 [9].

Over the past few years, bioprinting has garnered immense global interest, exerting a transformative influence on the field of biomedical sciences [10].

3D printing, broadly characterized as a process for translating a 3D model into a physical object, has garnered significant attention in recent times due to its expanding array of applications [11]. Bioprinting, on the other hand, is specifically defined as the precise deposition of biomaterials and living cells in a predetermined layer-by-layer arrangement to fabricate three-dimensional constructs [12]. In recent years, the evolution of diverse biomaterials has propelled the advancement of 3D printing, elevating it to the status of a novel engineering tool with the capacity to fabricate three-dimensional objects tailored to applications in the pharmaceutical sector. Furthermore, 3D bioprinting has gained widespread recognition as a methodology for constructing living tissues or organs, employing biological components such as cells and biomolecules [11].

Bioprinting offers several pivotal advantages, including reproducibility, precision, and the capability to generate intricate geometries [12,13,14]. It affords meticulous control over the spatial and temporal positioning of living cells, proteins, DNA, drugs, growth factors, and other bioactive substances, guiding the formation and development of tissues. The solutions containing biomaterials used in bioprinting are commonly referred to as “bioink” [15].

There are four primary categories of bioink materials employed in bioprinting, namely cell aggregates (comprising tissue spheroids, cell pellets, and tissue filaments), hydrogels, micro-carriers, and decellularized matrix components [16]. Similar to other biofabrication techniques, bioprinting can be executed through two fundamental approaches, scaffold-based and scaffold-free bioprinting [17]. Scaffold-based bioinks encompass cells embedded within decellularized extracellular matrix (ECM) constituents or hydrogels, where both natural and synthetic hydrogels find utility [18]. Scaffold-free bioinks consist of a high concentration of cells without the addition of supporting exogenous materials, encompassing tissue filaments, cell pellets, and spheroids, where cells autonomously generate their own ECM. The typical procedural steps in the bioprinting process encompass medical object (organ) imaging and computer-aided design model processing, bioink selection, bioprinting, and the subsequent in vitro or in vivo utilization of the bioprinted construct.

**Figure 2:**
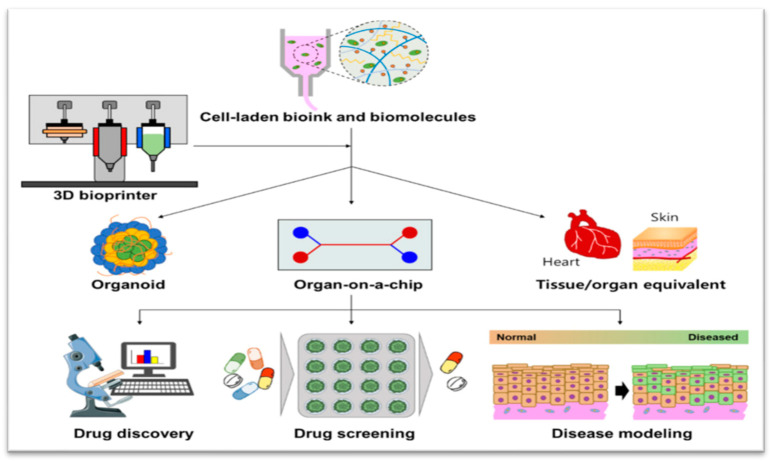
Schematic illustration representing 3D bioprinting enabling the creation of 3D cell culture devices, including organoid, Organ-on-a-Chip, and tissue/organ equivalent, which can enhance pharmaceutical applications for drug discovery, drug screening, and disease modeling [8].

The 3D bioprinting technique, as originally defined by Mironov et al., is characterized as the utilization of material transfer processes to pattern and assemble biologically relevant materials, including molecules, cells, tissues, and biodegradable biomaterials, in a predetermined organization with the aim of executing one or more biological functions [19]. This groundbreaking approach holds the potential to establish a novel standard method for the generation of biological models capable of emulating select functions of tissues and organs, particularly for the purposes of drug discovery and development, as shown in Figure 2 [8].

The 3D bioprinting process offers a distinct avenue for the assessment of pharmacokinetics, pharmacodynamics, and toxicology in preclinical studies, as well as for the exploration of phase I/II clinical trials in human subjects, marking a significant advancement in drug evaluation methodologies [20,21,22,23,24,25].

As such, 3D bioprinting has the potential to revolutionize drug trials, representing a departure from the conventional reliance on two-dimensional cytology, animal testing, and clinical trials. Organ-on-a-Chip technologies, which simulate the physiological characteristics of living organs (e.g., liver, heart), offer a promising alternative for drug testing, obviating the need for extensive animal experimentation [26,27,28,29].

Within the domain of 3D bioprinting, a few techniques are utilized, including laser-assisted bioprinting, stereolithography (SLA) bioprinting, jet-based bioprinting, and microextrusion bioprinting [30,31]. Among these, microextrusion bioprinting has shown great promise. Expulsion printing is a fundamental printing innovation that can print cell-laden structures under physiological conditions. Microextrusion printing is utilized to print gooey bio-inks and, in this way, gives a stage to print cell-laden structures proficiently and in a controllable way under physiological conditions [32]. Analysts frequently utilize bioinks composed of substances such as gelatin, hyaluronic corrosive, alginate, and decellularized extracellular framework (dECM) for the cell epitome, as these materials closely imitate the physical and chemical characteristics of the normal extracellular network (ECM), and have favorable traits such as biocompatibility, printability, mechanical astuteness, and biodegradability.

The cells utilized in 3D bioprinting mostly comprise stem cells, which can be categorized into different types such as mesenchymal stem cells, adipose-derived stem cells, and neural stem cells, as well as the pluripotent type. The innovation of 3D bioprinting allows for the exact and adaptable localization of these cells, empowering the designing of human tissues through excellent planning, well-developed bioinks, and effective polymerization strategies [33,34,35]. With similar progress, researchers have reported on a self-enhancing sonicated ink (or sono-ink), as well as a focused-ultrasound writing strategy for deep-penetration acoustic volumetric printing (DAVP) that allows for centimeter-deep printing within organic tissues, paving the way toward minimally invasive pharmaceutical interventions [36].

Through this approach, various tissues can be constructed utilizing a 3D bioprinter and bioink containing distinct cell types, with the possibility of subsequently transferring these tissues onto organs-on-chips. The automated nature of 3D printing facilitates the production of these Organ-on-a-Chip devices, composed of primary cells, gels, and other essential components. This is attributed to the inherent advantages of 3D bioprinting technology, which enables the precise control of cell distribution, extracellular matrix deposition, and the incorporation of biological materials, thereby facilitating the creation of Organ-on-a-Chip microfluidic devices [29].

In recent years, Organ-on-a-Chip (OoC) technology has emerged as a compelling alternative in drug development, as it aims to replicate the ecological, functional, and interconnected characteristics of human organs and tissues [37,38]. The concept of emulating the organic and physiological functions of the human body using cells within microfluidic chips was first introduced in the early 2000s by Shuler et al., who demonstrated a cell culture system that mimicked the interaction between the lung and the liver on a silicon chip no larger than a square inch. The term “Organ-on-a-Chip” was formally adopted in 2010 [34,39,40,41]. Compared to traditional models, this innovative technique provides enhanced insights into cell mechanical properties, morphology, and differentiation. OoC platforms facilitate the assessment of cellular responses, genetic expressions, and cellular functions [42]. These systems have a profound impact on the selection and evaluation of drug candidates, which are compounds, molecules, antibodies, etc., with significant therapeutic potential under investigation. They enable the collection of data on the metabolite secretion by specific cells when interacting with drugs [43]. OoC technology holds the promise of reducing and complementing the reliance on animals, cell models, and even human subjects in the development of new drugs [39]. It also serves as a valuable alternative in pharmacological, pharmacokinetic, and toxicological studies, thereby enhancing their efficacy, precision, and utility as research tools in drug development.

OoC systems offer a more profound understanding of pathophysiology, facilitating the design of new drugs targeting specific mechanisms and fostering therapeutic advances in conditions such as cancer, neurological diseases, and rare disorders [34]. For instance, Liu and colleagues investigated brain metastases using a multi-organ chip that exhibited functional barrier characteristics [44]. Choi and colleagues employed a three-dimensional (3D) tumor model to identify the most effective drug for combating lung cancer cells [45]. In a study of Gerbolés, the OBST (organoid-based frameworks for toxicological examination) procedure demonstrated a few points of interest for nanotoxicology/nanomedical examination as follows: cells can survive longer without sections; nanoparticles can spread and diffuse within the cell-laden multilayer by mirroring the in vivo introduction; and nanoparticles reach the 3D-printed cells in all layers with a discernible increase in internalization [46]. Amid the COVID-19 pandemic caused by SARS-CoV-2 in 2020, in the midst of a worldwide race to combat the disease’s movement, OoCs were considered for treatment purposes, giving rise to fast and dependable preclinical outcomes [47].

In the realm of pharmaceutical development, researchers face the task of selecting the appropriate bioprinting processes, including choosing bioinks tailored to specific pharmaceutical studies. Bioprinting has the potential to cater to the distinct requirements of various pharmaceutical investigations, such as in vitro predictive toxicology, high-throughput screening, drug delivery, and tissue-specific efficacy assessments.

Traditional pharmaceutical drug screening methods have heavily relied on 2D-based cell cultures and animal models to gain insights into the biological mechanisms of tested drugs and understand various disease states. However, these conventional screening models frequently fall short in faithfully representing the complexity of the human body, mainly due to differences in the microenvironments and genetic variations. The capacity of 3D bioprinting to generate living tissues that closely mimic native structures offers a promising solution to overcome the limitations of existing preclinical models. While pioneering research in drug screening has already demonstrated the advantages of 3D bioprinting technology, further exploration of patient-specific drug development is still in its initial stages. There is a need for more active research into various factors that may arise when utilizing patient-derived cells [48].

The integration of models with enhanced predictive capabilities during the preclinical phases can significantly reduce clinical failures [43]. While traditional two-dimensional (2D) cell cultures have made significant contributions to medicine and have reduced the dependence on laboratory animals, the animal models employed in preclinical phases often lack relevance to the human body. Consequently, they frequently fail to accurately predict drug efficacy and safety, underscoring the importance of advancing and adopting more physiologically relevant models [39,49,50,51,52].

Additive manufacturing (AM) processes, often referred to as three-dimensional printing (3D), involve the layer-by-layer deposition of material to generate objects from computer-aided design (CAD) files [53].

**Figure 3:**
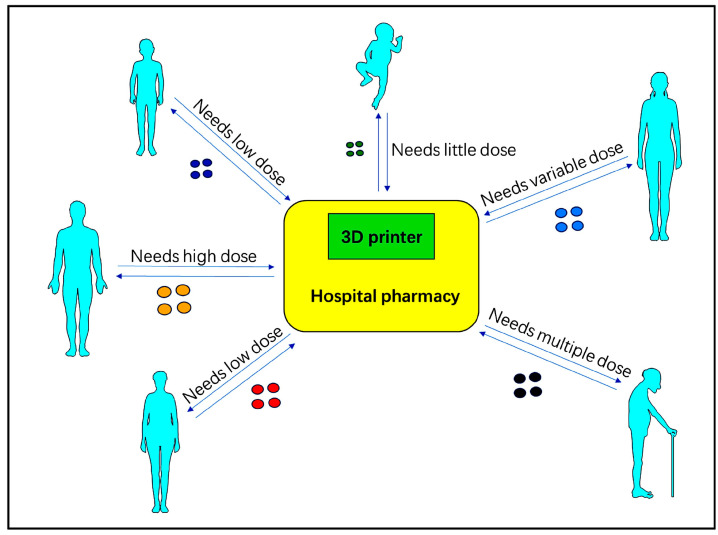
Based on the diagnosis, treatment, and treatment plan, the doctors can print the drug on the spot, adapting to the specific situation of each patient. The printed drugs should have a precisely measured API (Active Pharmaceutical Ingredient) [11].

Distinguishing between 3D printing and 3D bioprinting, we can summarize their differences as follows:

3D Printing: This technique involves the layer-by-layer construction of three-dimensional structures using various materials such as plastics, metals, polymer resins, and rubber. It is primarily utilized for producing a wide range of 3D objects. The purpose of producing high-quality 3D-printed items is to develop advanced materials and forms. Taking advantage of nanomaterials with tunable and unmistakable physical, chemical, and natural properties, integrating nanotechnology into 3D printing has given rise to unexplored possibilities for progressing 3D printing in the field of therapeutics. Recently, efforts have been made to make strides in restorative 3D printing through nanotechnology, providing knowledge into creating advanced restorative 3D printing innovations that have yet to be utilized [54]. In the medical field, 3D printing has been employed to create anatomical models, implants, prostheses, therapeutic devices, surgical instruments, specialized tools, and 3D models that aid surgeons in surgical planning and problem analysis. Successful clinical trials have even demonstrated its utility in preoperative planning [55]. In the pharmaceutical context, 3D printing can be used to fabricate drugs with complex structures to control release rates or to print custom-designed tablets for precise dosing. It also has the capability to produce intricate structures that can enhance drug absorption or reduce adverse reactions, as shown in Figure 3 [11].

3D Bioprinting: Bioprinting is based on the addition of substances (growth factors) and biomaterials to develop a microenvironment for living cells. These materials are regularly alluded to as bioinks and are based on cytocompatible hydrogels, which gel in a way consistent with distinctive bioprinting approaches [56]. This innovative technology is thus applied to create three-dimensional complex structures using living cells, biomaterials, and biological molecules [16,57]. Bioprinting operates similarly to standard 3D printing, with the key difference being the use of bioink instead of conventional ink. Bioink consists of cells and biomaterials required to form tissue constructs with a high level of repeatability, flexibility, and accuracy. Computer-driven bioprinters dispense cells and biomaterials with precision to create predetermined structures, typically through the following three stages: first, the collection of accurate tissue/organ information to select the appropriate materials and patterns; second, the transformation of this information into an electrical signal for printer control; and third, the development of the stable tissue/organ structure [58,59,60,61].

Three-dimensional printing of implantable microfluidic devices employing the support of a silicon composite could be a method that allows for effortless, fast, cost-effective, and high-resolution printing and presents the potential for the advancement of microfluidic gadgets for different applications such as, but not limited to, Organ-on-a-Chip gadgets, 3D bioprinting, and sedate testing [62].

3D bioprinting, as a subset of additive manufacturing of biomaterials, has wide-ranging applications in the pharmaceutical sector. It allows for precise control of cell and biomaterial distribution, enabling the construction of organoids or Organ-on-a-Chip models for drug testing. These models can mimic disease characteristics, replacing the need for animal testing and even certain clinical trials, including drug screening, drug testing, and research in areas like cancer and regenerative medicine [63].

Currently, major pharmaceutical companies, including Roche and AstraZeneca, are actively researching 3D bioprinting as a tool for creating preclinical study models. This underscores the significance of 3D bioprinting as an advanced and legitimate research approach in the pharmaceutical industry, with the potential to enhance drug development efficiency and reduce costs [64,65]. It represents one of the most cutting-edge and promising technologies in the field.

The objective of this review is to explore the latest advancements, accomplishments, and potential prospects associated with the application of 3D bioprinting technologies in the pharmaceutical domain.

## 2. Materials and Methods

The research adhered to the Preferred Reporting Items for Systematic Reviews and Meta-Analyses (PRISMA) guidelines for reporting meta-analyses and systematic reviews [66].

### 2.1. Literature Search

A comprehensive literature search was conducted using scientific databases, namely PubMed, Scopus, Google Scholar, and ScienceDirect. The search involved the use of specific keywords combined with Boolean operators (AND and OR). The keywords encompassed various aspects of 3D bioprinting as follows: additive manufacturing, drug research, personalized medicine, pharmaceutical companies, clinical trials, drug testing, Organ-on-Chip, drug delivery, drug screening, drug development, drug discovery, therapeutic strategies, drug-induced predictive toxicity screening, drug efficacy and safety, pharmacodynamics, pharmacokinetics, drug withdrawal, personalized treatment, pharmaceutical products, drug fabrications, drug dose structures, personalized drugs, pharmacoprinting in drug manufacturing, drug screening, pharmaceutical formulations, multi-drug embed, 3D printed drug, pharmaceutical manufacturing, personalized drugs, drug innovation, and drug development.

### 2.2. Eligibility Criteria

Study selection was based on specific eligibility criteria, incorporating both the inclusion and exclusion criteria. The inclusion criteria included articles published between 2019 and 2023, studies applying 3D printing and/or bioprinting in the pharmaceutical sector context, reviews, systematic reviews, or meta-analyses, and full-text articles. The exclusion criteria comprised abstracts, short communications, book chapters, case reports, and studies lacking fundamental information about bioprinting and the additive manufacturing process.

### 2.3. Data Analysis

Following the extraction of articles from the databases, a meticulous organization was carried out in an Excel sheet, eliminating any duplicates. Subsequently, four authors independently reviewed all the article abstracts. From the selected abstracts, the same authors independently read the full texts, facilitating a final selection of relevant studies. The results of the four authors were then compared and discussed until a consensus was reached.

## 3. Results

**Figure 4:**
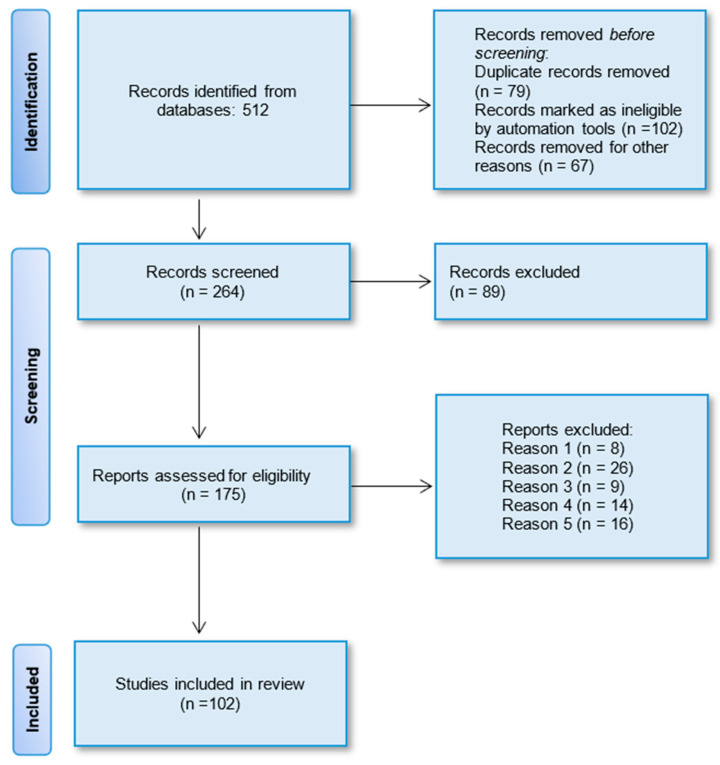
PRISMA flow diagram displaying the procedure of study selection.

As mentioned earlier, the authors adhered to the recommended guidelines of the Preferred Reporting Items for Systematic Reviews and Meta-Analyses (PRISMA) to conduct systematic reviews. Initially, 512 potentially relevant articles were identified from the four selected databases based on their titles. After removing duplicates, a total of 264 studies remained. Subsequently, through the evaluation of abstracts, 89 articles were excluded due to insufficient data and divergent study strategies. This left 175 full papers for a detailed analysis. Ultimately, 102 full-text articles were selected for this systematic review. The PRISMA flow chart illustrating the selection process of the studies is depicted in Figure 4.

Table 1 within the article offers a consolidated overview of the scientific literature, delineating the preeminent trajectories of bioprinting within the pharmaceutical domain. The table serves as a succinct compilation of crucial discoveries and research pathways, serving as a valuable point of reference for readers in search of a comprehensive understanding of the prevailing research landscape in this particular domain.

## 4. Discussion

Since the early 2000s, 3D bioprinting utilizing organic materials like cells and biomolecules has demonstrated its adequacy in tissue building. It allows for the coordinated creation of living structures that closely mirror the behavior of normal living structures [82]. This innovation is recognized for its potential in creating tissue stages with high reproducibility and adaptability by precisely integrating biomaterials with cells and other biomolecules. These properties of 3D bioprinting closely align with the prerequisites of drug screening and drug delivery procedures, thereby paving the way for more advanced pharmaceutical applications. For the meticulous design of physiologically functioning models and gadgets, researchers must consider the different factors of 3D bioprinting, including the determination of appropriate biomaterials, cell sources, and printing methodologies [9].

The numerous advantages of 3D printing and bioprinting are rapidly driving the advancement of pharmaceutical applications on multiple fronts. This progress encompasses the development and enhancement of existing drugs, new drug discovery, screening and testing systems, as well as the acceleration of drug approval and deployment timelines.

### 4.1. 3D Bioprinting in Drug Screening

Three-dimensional bioprinting plays a pivotal role in the identification and validation of drug targets throughout the drug discovery and development process. As such, 3D-bioprinted disease models are instrumental in high-throughput screening for in vitro efficacy, representing a crucial step toward successful drug development [67]. Moreover, in the optimization of potential drug candidates, 3D-bioprinted constructs serve as valuable tools for evaluating the in vitro efficacy and retrospective toxicity assessments, following the identification of target organs from in vivo toxicity tests. Through a continuous refinement process involving the exploration of structure–activity-specific relationships, new compound synthesis, and a series of in vitro and in vivo assays, potential drugs can be selected from a pool of optimized drug candidates, eventually undergoing regulatory testing before reaching the market [1,8,69,70].

To effectively evaluate the pharmaceutical safety and therapeutic efficacy of drug candidates, in vitro analytical platforms must accurately replicate the anatomical features and critical functions of target tissues and organs. Leveraging the precise positioning of cells, biomolecules, and biomaterials, 3D bioprinting techniques enable the creation of advanced 3D cell culture devices, typically categorized into three common entities as follows: organoids, Organ-on-a-Chip (OOC) systems, and tissue/organ equivalents. Drug screening also offers substantial time and cost savings by swiftly eliminating unsuitable candidates during the initial validation stages. Moreover, the importance of screening to differentiate drugs suitable for effective therapeutic applications continues to grow [9].

### 4.2. Use of 3D Bioprinting in Preclinical Drug Trials

Introducing a new drug to the market is an expensive and time-consuming process. Drug development costs can range from $161 million to $4.54 billion, and it typically takes around 12–15 years to obtain FDA approval [83,84]. The successful commercialization of a drug necessitates significant investments of time and financial resources, and failures in later stages of drug development can result in substantial setbacks. Therefore, there is a constant need for innovative technologies that can reliably predict the efficacy and toxicity of drug candidates early in the drug discovery process before proceeding to clinical trials [8].

Preclinical trials constitute a significant portion of the total capital costs, accounting for an average of 42.9%. However, the failure rate for drug candidates that advance to Phase I clinical trials is a staggering 90% [85,86]. Preclinical studies encompass in silico, in vitro, and in vivo models. While in vivo testing is crucial for safety assessment, it carries several drawbacks, including the need for approval from animal ethics committees, high research costs, challenges in replicating human physiology, and lengthy protocols [87,88,89,90,91,92]. The ethical dilemma of using animals in preclinical testing has been a longstanding concern in pharmaceutics and medicine [93]. The question of improving the welfare of animals used in scientific research, known as the 3Rs (replacement, reduction, and refinement), was raised by researchers in the mid-twentieth century and continues to be relevant today [94,95]. The use of animals in preclinical testing has increased with advancements in drug technology and research and development [93].

Bioprinting is poised to become a significant tool in the global movement to replace animal experiments with more advanced in vitro models for studying disease mechanisms and conducting high-throughput pharmacological and toxicological assays [69].

### 4.3. 3D Bioprinting’s Impact on Drug Development and Testing

Researchers have been harnessing the power of 3D bioprinting to test drugs using organ models. For instance, companies like Organovo, a bioprinting firm, and Roche, a pharmaceutical giant, have utilized 3D-printed “livers” to evaluate drug toxicity at various levels and detect liver damage caused by drugs like trovafloxacin [64]. In another example, researchers created a 3D-printed brain mini-model containing both glioblastoma multiforme (GBM) and glioblastoma-associated macrophages (GAMs) to simulate the interactions between these cell types and test the efficacy of anti-tumor drugs [71].

The precision of 3D printing in creating specific geometric shapes allows for personalized drug formulation, including the drug’s form and dosage. Furthermore, 3D printing overcomes the limitations of traditional formulation techniques by tailoring tablet microstructure and altering composition, enabling the production of tablets with distinct release profiles [96,97,98]. The integration of Organ-on-a-Chip technology into 3D bioprinting ensures the precise distribution of cells, extracellular matrix, biomaterials, and circulatory systems within these models [72,73,98]. When combined with 3D printing technology, bioprinting can enhance the effectiveness of drug delivery devices. A clear example of the commercial viability of 3D printing in drug production is Spritam, a drug product manufactured by Aprecia Pharma, Inc. It has received clearance for sale and use by the US FDA [72]. Although 3D printing technology faces some challenges related to methodologies and materials, these issues are expected to be resolved as technology advances. The future impact of drug testing on patients is set to be significantly enhanced, with more pharmacies and hospitals equipped with 3D printing facilities for on-demand personalized pharmaceutics solutions [73].

### 4.4. Advantages of Organ-on-a-Chip Technology as a Pharmaceutical Platform

Organ-on-a-Chip technology holds greater potential than traditional cell cultures for accurately predicting functional impairments, adverse effects, pharmacokinetics, toxicological profiles, and drug efficacy. This potential arises from the capability to arrange different cell lines within a three-dimensionally structured polymer and simulate and control specific conditions. It allows for the emulation of normal organ, tissue, barrier, or physiological processes, as well as the interaction between different systems. Additionally, this approach enables the study of physiological and pathophysiological processes, bridging multiple disciplines like materials engineering, cell cultures, and physiology [42].

The occurrence of adverse drug reactions is a significant challenge in drug development and one of the key reasons for drug failures in preclinical models as predictors of clinical outcomes [37,49,50,99]. Clinical failures can result from safety issues, as well as the lack of clinical efficacy [100]. Inadequate assessment of adverse effects can also become a problem for a drug that has already advanced to the clinical phase, potentially leading to its withdrawal from the market. Adverse events are often associated with organs like the liver or heart due to the generation of metabolites [39,99].

Bhise et al. [27] created a Liver-on-a-Chip platform to test drug toxicity through 3D bioprinting and HepG2/C3A cells. The release rate of four biomarkers (egg whites, alpha-1 antitrypsin, transferrin and ceruloplasmin) continuously increased throughout the study, expanding with the number of cells until day 30, showing the great work of the liver structure. At that point, they underwent an intense hepatotoxic reaction with acetaminophen (APAP). In a bioreactor without APAP treatment, the metabolic action increased by 4%; within a bioreactor treated with APAP, the metabolic action diminished by 2%. This suggests that the Liver-on-a-Chip reacts to intensely harmful drugs and can be utilized for drug screening. Zhang et al. [28] depicted in their study, a strategy for producing endothelialized organoids created through 3D bioprinting, which may allow for a wide array of applications in regenerative medicine, drug screening, and potential infection modeling. They proposed a novel 3D bioprinting-based cross-linking methodology to produce endothelialized myocardium, employing a composite bioink with endothelial cells directly bioprinted into hydrogel scaffolds which then migrated to the outskirts of the microfibers to create a layer of intersecting endothelium. At that point, the 3D endothelial scaffold is “seeded” with cardiomyocytes to produce a myocardium capable of unrestricted and simultaneous removal. Finally, they implanted the organoids in a custom-designed microfluidic perfusion bioreactor to complete the endothelialized Myocardium-on-a-Chip stage for analyzing cardiovascular toxicity.

### 4.5. Application of 3D Bioprinting in Skin-Related Research

Human bioengineered skin substitutes can be utilized for different clinical and investigate applications [74,75,76,101,102]. With the growing interest in cosmetic/aesthetic strategies and increasing rates of weight, diabetes, and a maturing population, the recovery of damaged or lost tissue has become a global issue, and the demand for skin biomanufacturing is continuously increasing. It is suggested that bioprinted skin allows for an elective approach to clinical applications in regenerative medication (inveterate wounds, burns, ulcerations, reconstructive surgery after major oncological resections); the modeling of physiological/pathological conditions (wound healing, UV reactions, aging, skin layer penetrability, drug reactions, photoirradiation, skin cancer, genodermatoses, burn conditions); and in the cosmetic/pharmaceutical industry (safety and efficacy of active substances, drug retention, drug metabolism, personalized treatments) [103]. Moreover, bioprinted skin models can serve as a platform for creating modern drug formulations [77]. Certain legislations, ethical directions, and moral reasons related to the safety and viability testing of modern formulations on animal models from the cosmetic and pharmaceutical sector necessitate investigations into under-utilized processes within the field of cosmetology and pharmaceutics [78]. Ex vivo skin production could be a valuable consideration within skin penetration studies, but due to financial and practical limitations, the advancement of alternatives is vital. On the other hand, conventional 2D cell culture has critical restrictions, which is why innovative advancements such as 3D bioprinting are required [55].

### 4.6. 3D Bioprinting in Drug Personalization and Cancer Treatment

Bioprinted tissues/organs can be of great benefit in leading drug candidate prioritization, toxicity testing, and disease/tumor models. Bioprinted tissues/organs can facilitate the testing of new compounds or the prediction of toxicity, as the spatial and chemical complexity characteristic of natural options can be recreated [79]. Additive manufacturing in terms of drug printing may also indicate an innovative technique in the production of patient-specific drugs and in customizing the formulation and dosage required by patients. Drug printing raises the idea of personalized drugs, making them safer and more effective [2,104].

Researchers at Columbia College within the U.S. have made a notable breakthrough with the advancement of a multi-organ chip which is exceptionally small in size and can be utilized to evaluate the impact of cancer drugs on the body [105]. Their discoveries were distributed in the journal, Nature. The chip comprises constructed tissues, including the heart, bones, liver and skin, which are associated by blood vessels to safe circulating cells, creating limited organ capacities. By testing the impact of the widely used cancer drug, Daunorubicin, on the heart, liver, bones, skin and blood vessels, the researchers were able to precisely predict its metabolism and dissemination within the chip. The outcomes of the multi-organ chip are comparable to those of other clinical trials, making it a valuable tool to improving cancer treatments. They have also been demonstrated to be customizable, focused on the treatment of individual patients [80].

Shinha et al. utilized a multi-organ chip, PK-PD, to assess the impact of simvastatin and ritonavir on the metabolism of CPT-11, an anticancer “prodrug” [106]. By observing the part of the liver involved in drug metabolism and the reaction of the cancer cells to the drug, the PK-PD was able to calculate particular parameters that allowed for the assessment of the drug’s effectiveness.

Multi-organ chips are important devices for analyzing drug capacity and determining potential harmfulness prior to human trials. The use of PK-PD models provides an understanding into the components of such factors and may encourage the development of more effective and safer drugs. These innovative strategies may lead to advanced drug evaluations and personalized pharmaceutical approaches in the future [80].

The application of tissue-specific models for bioprinting organs or specific tissues supports the testing of therapeutic regimens and clinical diagnosis, as well as the treatment of disease by replacing damaged tissues [67]. It will be possible to predetermine personalized pathophysiological states by taking into account the genome, proteins, and medical/family history, and therefore limit life-threatening effects [107].

Nowadays, 3D bioprinting helps laboratories scale up the production of organoids at a rate of thousands per hour to test the effects of infectious diseases and drug therapies. In order to recreate the environment of the native organ, scientists also use devices called Organs-on-a-Chip [108]. The next scientific step is to develop multiple Organs-on-a-Chip that are combined into an end-to-end system. For example, a cancer drug can be screened through a Tumor-on-a-Chip that is connected to healthy Tissue-on-a-Chip to find out if the treatment dose shrinks the tumor without damaging the healthy tissue [109].

Cutting-edge translational cancer research might be beneficial to the study of human tissue-based models. In any case, creating an environment where these models can be utilized to curb a few considerations will be challenge for researchers. Increasing the application of human tissue models will require investigation into the foundation and preparations required, as well as the execution of “de-risking” methodologies for achieving modern models, including benchmarking and information sharing [110].

A few researchers have considered so-called “keen” quality production facilities with high-tech, sterile clean rooms. Custom 3D bioprinted tissues and Organ-on-a-Chip gadgets can be made by pharmaceutical companies thus helping to realize efficient testing with more significant novelty and precision. Oncologists may also send the individual patient’s cancer tissues and drug details to the manufacturing plant to be tested on 3D-fabricated chip gadgets [111].

Bioprinting can be utilized as a viable tool to progress preclinical studies within the near future. There is still much room for advancement in terms of biomaterial arrangement and fine-tuning of the bioprinting process itself. Machine learning can be utilized as a compelling tool for this, as it has already been demonstrated as a strategy for cell quality control [112].

Bioprinting can precisely control the dissemination of cells, dynamic atoms, and biomaterials. In addition, this innovation summarizes key highlights of the tumor microenvironment and develops in vitro tumor models with bionic structures and physiological frameworks. These models can be utilized as vigorous stages to consider tumor initiation, interaction with the microenvironment, angiogenesis, motility and intrusion, as well as intra- and extravasation. Bioprinted tumor models can also be utilized for high-throughput drug screening and approval and encourage personalized cancer treatment investigations [81].

Currently, bioprinting is a rapidly developing field of biomaterial engineering. With opportunities to achieve scientific breakthroughs, bioprinting technology has found its place in preclinical studies, which will rapidly push the pharmaceutical and biomedical industries forward in the future [93].

The generation of modern drug formulations utilizing 3D printing is anticipated to play a crucial role within the advancement and development of different drug delivery frameworks. Different from the traditional pharmaceutical approaches that rely on mass generation, 3D printing approaches can possibly give rise to customizable medicine, improving on the effects and side effects of drugs for individual patients. Studies into 3D-printed drug delivery systems currently under examination are based on the analysis of drug release and drug delivery; thus, 3D printing is an innovative and compelling approach to gaining an understanding of patient-specific drug delivery systems [9].

## 5. Limitations and Future Directions

Drug bioprinting, an innovative approach combining 3D printing with pharmaceutical sciences, offers substantial potential for personalized medicine, drug development, and customized drug delivery systems. However, this field faces several limitations and offers exciting directions for future research and development.

### 5.1. Limitations of Drug Bioprinting

Regulatory Challenges

⮚ Regulatory frameworks for bioprinted drugs are still under development. Ensuring safety, efficacy, and quality control poses significant challenges.
⮚ Standardization and validation of bioprinting processes need comprehensive guidelines to gain regulatory approval.

Technical Limitations

⮚ Resolution and precision of bioprinting technologies are not yet optimal for creating complex drug formulations.
⮚ Scalability is a significant issue. Current bioprinting techniques are more suited for small-scale, personalized applications rather than mass production.

Material Limitations

⮚ Limited availability of biocompatible and bioactive materials that can be used for drug printing.
⮚ Stability of printed drugs during storage and transportation is a concern, as some materials may degrade over time.

Complexity of Formulations

⮚ Difficulty in printing multi-drug combinations with precise control over dosage and release profiles.
⮚ Achieving uniformity and consistency in drug distribution within the printed matrix can be challenging.

Cost

⮚ High initial setup costs for bioprinting equipment and materials.
⮚ Cost-effectiveness of bioprinted drugs compared to traditional manufacturing methods needs to be evaluated.

Biological Interactions

⮚ Understanding the interactions between bioprinted materials and biological systems is still in the early stages.
⮚ Potential immune responses or toxicological effects of new bioprinted drug formulations need thorough investigation.

### 5.2. Future Directions in Drug Bioprinting

Advanced Materials

⮚ Development of new biocompatible and bioactive materials that can improve the functionality and stability of bioprinted drugs.
⮚ Exploration of natural and synthetic polymers, hydrogels, and composite materials to enhance drug delivery systems.

Technological Innovations

⮚ Improvement in printing technologies to achieve higher resolution, precision, and scalability.
⮚ Integration of real-time monitoring and feedback systems to ensure quality control during the printing process.

Personalized Medicine

⮚ Expansion of bioprinting applications to create patient-specific drug formulations tailored to individual needs.
⮚ Use of patient data and AI-driven design for optimizing drug formulations and delivery systems.

Combination Therapies

⮚ Research into multi-drug printing capabilities to develop complex drug delivery systems that can administer multiple drugs in a controlled manner.
⮚ Innovations in spatial and temporal control of drug release profiles.

Regulatory Frameworks

⮚ Development of comprehensive regulatory guidelines specific to bioprinted drugs to ensure safety and efficacy.
⮚ Collaboration between industry, academia, and regulatory bodies to create standardized protocols and validation methods.

Clinical Translation

⮚ Conducting more clinical trials to validate the efficacy and safety of bioprinted drugs in real-world scenarios.
⮚ Collaboration with healthcare providers to integrate bioprinting technologies into clinical practice.

Sustainability

⮚ Exploration of sustainable materials and processes to reduce the environmental impact of bioprinting.
⮚ Development of reusable or biodegradable bioprinting components.

Interdisciplinary Collaboration

⮚ Fostering collaboration between materials scientists, biologists, engineers, and pharmacologists to address the multifaceted challenges of drug bioprinting.
⮚ Encouraging interdisciplinary research and development to push the boundaries of what is possible with bioprinting technologies.

In summary, while drug bioprinting faces significant limitations, ongoing research and technological advancements hold great promise for overcoming these challenges and revolutionizing the field of pharmaceutical sciences.

## 6. Conclusions

3D bioprinting indeed holds significant promise in the field of pharmaceutics, especially when it comes to drug development, personalized medicine, drug screening, and testing. Here is a summary of its applications and benefits:

Drug Development: 3D bioprinting can be employed in the development of new drug molecules. It allows for the precise placement of cells and biomaterials, enabling the creation of complex tissue models for testing drug candidates.

Personalized Medicines: Bioprinting offers the potential for personalized medicine by tailoring drug formulations and delivery systems to individual patient needs. This can enhance treatment efficacy and reduce side effects.

Drug Screening and Testing: One of the primary applications of bioprinting is in drug screening and testing. It allows for the creation of organoids and Organ-on-a-Chip models that mimic disease characteristics. These models can replace animal testing in preclinical trials, providing more accurate and relevant results.

Customized Drug Screening Platforms: Bioprinting technology enables the development of customized drug screening platforms, allowing researchers to study drug responses in tissue-specific contexts. This is valuable for assessing drug efficacy and toxicity.

Shortening Time to Market: By facilitating more accurate and efficient preclinical and clinical drug testing, bioprinting can contribute to shortening the time it takes to bring a drug product to market. This can potentially reduce the overall cost of drug development.

Overall, 3D bioprinting has the potential to revolutionize drug development and testing processes, making them more efficient, cost-effective, and tailored to individual patient needs. It aligns with the growing emphasis on precision medicine and the reduction in animal testing in pharmaceutical research.

## Figures and Tables

**Table 1:** The most significant directions of bioprinting in the pharmaceutical sector.

Key Benefit/Topic	Area of Application/Significance	References
3D Bioprinting in Drug Screening	Crucial step toward drug development success	(Shopova et al., 2023) [67]
	3D bioprinted constructs serve as valuable tools for evaluating in vitro efficacy and retrospective toxicity	(Bom et al., 2021) [1], (Peng et al., 2017) [8] (Moldovan, 2021) [68] (Aquino et al., 2018) [69] (Koçak et al., 2021) [70]
Use of 3D Bioprinting in Preclinical Drug Trials	3D Bioprinting technologies can reliably predict the efficacy and toxicity of drug candidates early in the drug discovery process	(Peng et al., 2017) [8]
	Bioprinting is poised to become a significant tool in the global movement to replace animal experiments	(Moldovan, 2021) [68]
3D Bioprinting’s Impact on Drug Development and Testing	3D bioprinting has power to test drugs using organ models	(Heinrich et al., 2019) [71]
	3D printing overcomes the limitations of traditional formulation techniques	(Okkalidis and Marinakis, 2020) [72]
	Bioprinting can enhance the effectiveness of drug delivery devices	(Chakka and Salem, 2019) [73]
Advantages of Organ-on-a-Chip Technology as a pharmaceutical platform	Organ-on-a-Chip technology holds greater potential for accurately predicting functional impairments, adverse effects, pharmacokinetics, toxicological profiles, and drug efficacy	(Vargas et al., 2019) [42]
	Innovative 3D bioprinting technology may find wide application in regenerative medicine, drug screening, and potential disease modeling	(Zhang et al., 2016) [28]
Application of 3D bioprinting in skin-related researches	Human bioengineered skin substitutes can be used for various clinical and research applications	(Sarkiri et al., 2019) [74] (Tavakoli and Klar., 2021) [75] (Smandri et al., 2020) [76]
	Bioprinted skin models can serve as a platform for developing new drug formulations	(Yan et al., 2018) [77]
	Bioprinting is new solutions in the field of cosmetology, pharmaceutics and medicine	(Millas et al., 2019) [78]
3D Bioprinting in Drug Personalization and Cancer Treatment	Bioprinted tissues/organs can be of great benefit in leading drug candidate prioritization, toxicity testing, and disease/tumor models	(Van Daal et al., 2020) [79]
	Drug printing raises the idea of personalized drugs, making them safer and more effective	(Aimar et al.) [2]
	Multi-organ chips are valuable tools for analyzing drug interactions and identifying potential toxicity prior to human trials	(Qu et al., 2023) [80]
	The application of tissue-specific models for bioprinting organs or specific tissues supports the testing of therapeutic regimens and clinical diagnosis	(Shopova et al., 2023) [67]
	Bioprinted tumor models can also be used for high-throughput drug screening and validation and enable personalized cancer treatment research	(Mao et al., 2020) [81]
	3D printing technology is an effective approach for the formulation of patient-specific drug delivery systems	(Gao et al., 2021) [9]

## Data Availability

Not applicable.
